# Color Doppler ultrasonography as an alternative tool for postoperative evaluation of collaterals after indirect revascularization surgery in Moyamoya disease

**DOI:** 10.1371/journal.pone.0188948

**Published:** 2017-12-08

**Authors:** Shin-Joe Yeh, Sung-Chun Tang, Li-Kai Tsai, Chung-Wei Lee, Ya-Fang Chen, Hon-Man Liu, Shih-Hung Yang, Meng-Fai Kuo, Jiann-Shing Jeng

**Affiliations:** 1 Stroke Center and Department of Neurology, National Taiwan University Hospital, Taipei, Taiwan; 2 Department of Medical Imaging, National Taiwan University Hospital, Taipei, Taiwan; 3 Department of Neurosurgery, National Taiwan University Hospital, Taipei, Taiwan; University of Rome, ITALY

## Abstract

The cerebral hypoperfusion caused by chronic progressive stenosis or occlusion of intracranial arteries in moyamoya disease can be treated by direct bypass or indirect revascularization procedures. The extent of collaterals from the external carotid artery (ECA) after indirect revascularization surgery is the key point of angiographic follow-up, and the invasiveness of angiography impelled us to investigate the role of ultrasonography in the evaluation of collaterals. We hypothesized that the collaterals shown on angiography might produce corresponding hemodynamic changes in color Doppler ultrasonography. We prospectively recruited moyamoya patients who underwent indirect revascularization surgery and received both preoperative and postoperative angiography and color Doppler ultrasound studies. The collaterals on angiography were graded according to Matsushima method. A total of 21 patients (age, 17 ± 10.2 years) with 24 operated hemispheres were enrolled. Patients who showed better collateral establishment by angiography had higher end-diastolic velocity (EDV), lower resistance index (RI), and larger flow volume in the superficial temporal artery (STA) and ECA (all p < 0.05). In STA, increase of EDV greater than 13.5 cm/sec or reduction of RI greater than 0.19 after operation corresponded to 94% of Matsushima grade A+B. In ECA, post-operative EDV greater than 22 cm/sec or increase of EDV greater than 6.4 cm/sec also corresponded to 94% of Matsushima grade A+B. Our findings revealed potential roles of color Doppler ultrasonography in identifying patients with poor collaterals after indirect revascularization procedures.

## Introduction

Moyamoya disease (MMD) is an important etiology among pediatric as well as adult stroke patients especially in Far Eastern countries [1–3]. Ischemic stroke is prevalent in both age groups of MMD, while haemorrhagic stroke is presented more frequently in adult than in pediatric moyamoya patients [2]. MMD is characterized by chronic progressive stenosis or occlusion of intracranial arteries, which leads to cerebral hypoperfusion in bilateral cerebral hemispheres [1,4]. The cerebral hypoperfusion of MMD can be treated by extracranial-intracranial bypass surgeries, including direct bypass, indirect revascularization, and combined surgeries [5]. The procedures of indirect revascularization surgeries use superficial temporal artery (STA), temporalis muscle, periosteum, or dura as graft in encephalo-duro-arterio-synangiosis (EDAS), encephalo-myo-synangiosis (EMS), encephalo-periosteo-synangiosis (EPS), and multiple burr hole (MBH) surgeries, respectively [6]. After indirect revascularization procedures, transpial and transdural collaterals or neovascularization establish gradually from the graft supplied by superficial temporal artery (STA) and external carotid artery (ECA) with corresponding improvement in the cerebral blood flow (CBF) shown on angiography and MRI [6–8], but there are differences among patients in growth speed and extent of the neovascularization. Thus, follow-up for the extent of collateral development is crucial after indirect revascularization surgeries.

Cerebral angiography is the gold standard tool for follow-up after indirect revascularization surgery in moyamoya patients, and the collaterals supplied by ECA is graded according to the criteria proposed by Matsushima et al. [9]. However, the invasiveness and radiation exposure of the procedure impede regular angiographic examination especially in pediatric patients [10]. Ultrasonography is a noninvasive tool for evaluation of blood flow in many clinical conditions. In moyamoya patients before operation, lower resistance in the STA has been revealed by color Doppler ultrasonography, which is compatible with the existence of collateral flow from STA to the intracranial hypoperfusion regions [11]. In a case report of a moyamoya patient presenting with non-aneurysmal subarachnoid hemorrhage (SAH), serial transcranial color Doppler examinations and cerebral angiography during resolution of SAH showed that the consistently high flow velocity and low resistance in the intracranial arteries except for stenotic arteries represented the hemodynamic influence by the collateral network of MMD [12]. After indirect revascularization surgery, only two studies used power Doppler ultrasonography in quantification of collaterals [10, 13], but the usefulness of color Doppler ultrasonography in evaluation of the post-operative neovascularization remains unclear. We hypothesized that the post-operative collaterals might produce corresponding hemodynamic changes in color Doppler ultrasonography. This study aimed to investigate color Doppler ultrasonography as a follow-up tool by correlating the ultrasonographic parameters with angiographic Matsushima grades in moyamoya patients after indirect revascularization surgery. In this study, STA was measured in addition to routine examination since STA was the major arterial supplier of EDAS and EMS, which are common procedures of indirect revascularization [6].

## Materials and methods

### Patients

Patients who were newly diagnosed with MMD by cerebral angiography and who received indirect revascularization surgery between August 2012 and December 2015, were prospectively registered in the current study. The indirect revascularization procedures included EDAS, EMS, EPS or multiple craniotomies (MC). All of these surgeries used STA as a material for revascularization. To compare the post-operative results between angiography and carotid ultrasonography, the inclusion criteria were that both angiography and carotid ultrasonography were performed before and after surgery, and the interval between the two post-operative imaging studies of individual patient was within 4 days. This study was approved by the Institutional Ethics Committee of National Taiwan University Hospital, and written informed consent was obtained from all enrolled adult patients, or from the parents of pediatric patients.

### Evaluation of collateral establishment by cerebral angiography

Cerebral angiography was performed for preoperative diagnosis and postoperative evaluation of the transdural and transpial collaterals at the synangiosis site in all patients. The timing of post-operative angiography varied depending on the clinical condition of the individual patient. The collaterals at the site of revascularization shown by injection of contrast from ECA were considered grade A if they supplied greater than two-thirds of the MCA territory, grade B if they supplied between one-third and two-thirds of the MCA territory, and grade C if they supplied less than one-third of the MCA territory [9]. The angiograms were evaluated by two independent observers and the results were determined consensually.

### Ultrasonographic evaluation

We performed the ultrasound study by using a color-coded ultrasound system (Philips medical system, IE33, Washington, USA) with 11–3 MHz linear-array transducer to examine extracranial arteries (carotid Doppler ultrasound) and 5–1 MHz phased-array probe to examine intracranial arteries (transcranial color-coded sonography, TCCS). The ultrasound examinations were performed by the same neurological ultrasound technician who was blind to the results of cerebral angiography. The parameters for extracranial arteries included peak-systolic velocity (PSV), end-diastolic velocity (EDV), resistance index (RI), and flow volume (FV) of the common carotid artery (CCA), ICA, ECA, and STA. The STA was analysed at the common STA segment at the level of the ear [14]. The flow volume was calculated automatically by the ultrasound machine from the time-averaged mean flow velocity with the cross-sectional area of the individual vessel, which was acquired at the end-diastolic point to minimize variation of the diameter. Pulsatility index (PI) was calculated as dividing (PSV-EDV) by time averaged mean velocity. For intracranial arteries, the measured parameters included PSV, mean velocity, and PI, and the results of anterior cerebral artery (ACA), MCA, and posterior cerebral artery (PCA) were recorded for this analysis. The data of ultrasonographic parameters were recorded exactly according to the examination results, and the data acquisition was blind to the angiographic results.

### Statistical analysis

The patients were classified into three groups according to the Matsushima grades on the post-operative angiography. Categorical variables are presented as percentages and continuous variables as mean ± standard deviation. In the analysis of baseline demographic data, the Kruskal-Wallis test was used for comparison of medians among groups with different Matsushima grades, and the Fisher’s exact test was used for analysis of categorical variables between groups. Second, we compared the hemodynamic parameters on post-operative ultrasound study between different Matsushima groups. Third, the changes of the ultrasonographic parameters between pre- and post-operative state were also compared between different Matsushima groups. The aforementioned two comparisons were analysed by Kruskal-Wallis test and Mann-Whitney two-sample rank sum test. Logistic regression analysis was used to investigate the correlation between Matsushima grades and ultrasonographic parameters. We also used Receiver Operating Characteristics (ROC) analysis of the ultrasonographic parameters to decide cut-off points according to Youden’s index. Two-tailed *P* values of less than 0.05 were considered statistically significant. Data management and analysis were performed using Small Stata software (StataCorp LP, College Station, Texas, USA).

## Results

There were 21 patients (male/female = 8/13) aged 5–41 years (mean, 17 ± 10.2 years) with a total of 24 surgically treated hemispheres in this study. The baseline demographic data are shown in Table 1. All of these patients had bilateral MMD findings. The post-operative Matsushima grade was A in 11 hemispheres, B in 7, and C in 6 with a follow up period of 8.5 ± 7 months (range, 1–24 months). The medians of pre-operative Suzuki stages were 3, 3, 2 in Matsushima A, B and C groups, respectively. The mean interval between post-operative angiographic and ultrasonographic studies was 1.8 ± 1.3 days (range, 0–4 days). About the operative procedures, 13 hemispheres underwent EDAS surgery, while another 7 hemispheres received EDAS combined with EPS, 1 hemisphere received EDAS combined with EMS, and 3 hemispheres underwent MC surgery. There was no statistical difference in age, sex, and follow-up interval among groups with different Matsushima grades.

**Table 1:** Basic demographics of the study subjects.

	Matsushima grade			
	A	B	C	P-value
Hemisphere number	11	7	6	
Case number	10	6	5	
Male	3	3	2	0.845
Age (mean ± SD), years	15.5 ± 9.9	15.8 ± 7.2	23.3 ± 13	0.704
Suzuki stage (median)	3	3	2	0.283
Postoperative follow-up duration (mean ± SD), months	7.7 ± 7.1	9.6 ± 8.2	8.5 ± 5.0	0.687
Surgical procedures				0.166
EDAS	5	6	2	
EDAS+EPS	5	0	2	
EDAS+EMS	0	0	1	
MC	1	1	1	

EDAS, encephalo-duro-arterio-synangiosis; EPS, encephalo-periosteo-synangiosis; MC, multiple craniotomies; EMS, encephalo-myo-synangiosis.

**Fig 1:**
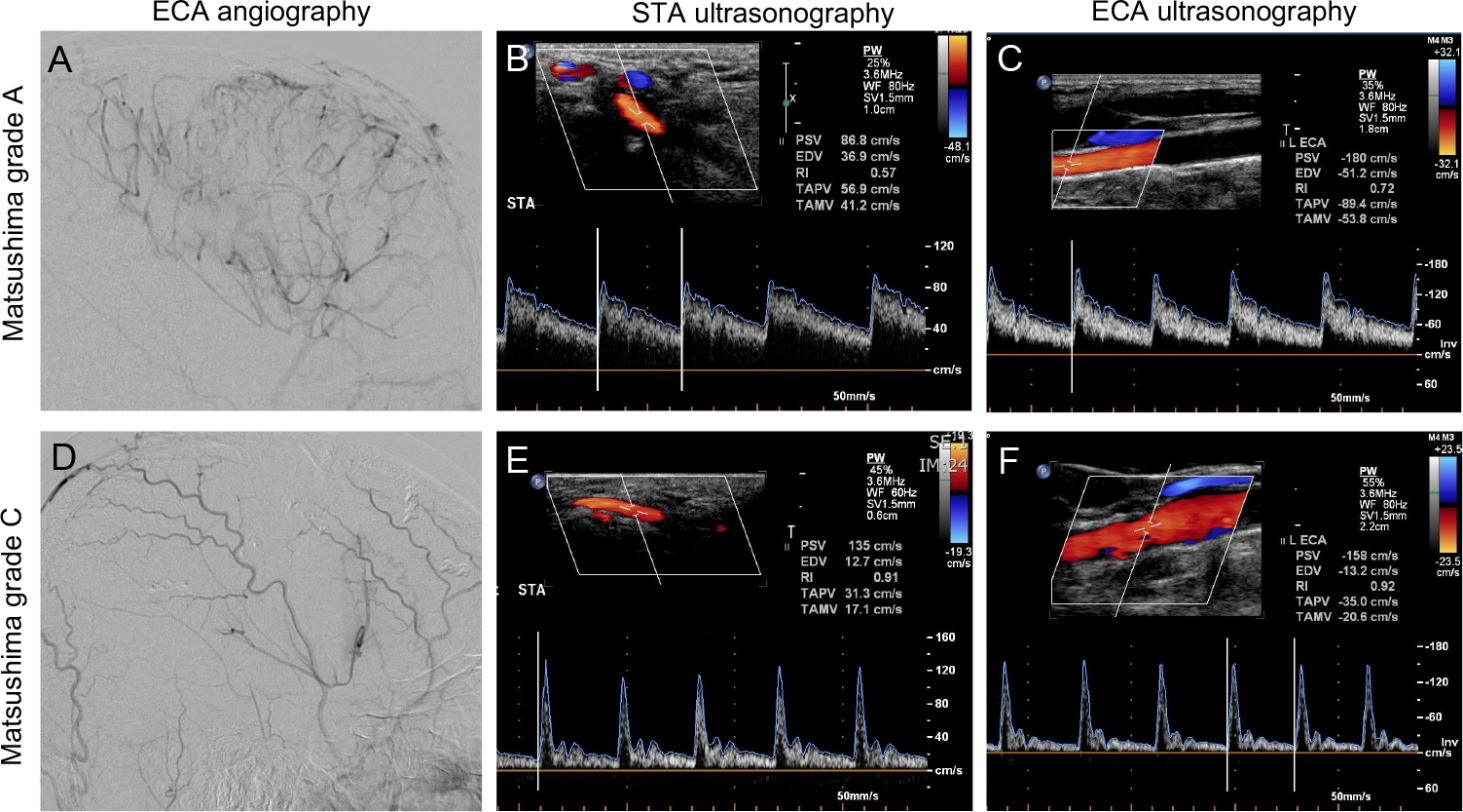
Postoperative angiogram and ultrasonographic flow patterns of ipsilateral STA and ECA in two representative cases. Case 1: Well-developed collateralization on ECA angiogram (Fig 1A) and reduced resistance in the STA (Fig 1B) and ECA (Fig 1C) on ultrasound. Case 2: Poor collateral establishment on ECA angiogram (Fig 1D) and ultrasound showed high resistance pattern in the STA (Fig 1E) and ECA (Fig 1F).

The post-operative angiographic and ultrasonographic patterns in two representative cases were shown in Fig 1. The first patient with Matsushima grade A on angiography had a low resistant flow pattern in STA and ECA on ultrasonography, while the second patient with Matsushima grade C showed a high resistant flow pattern in STA and ECA.

**Fig 2:**
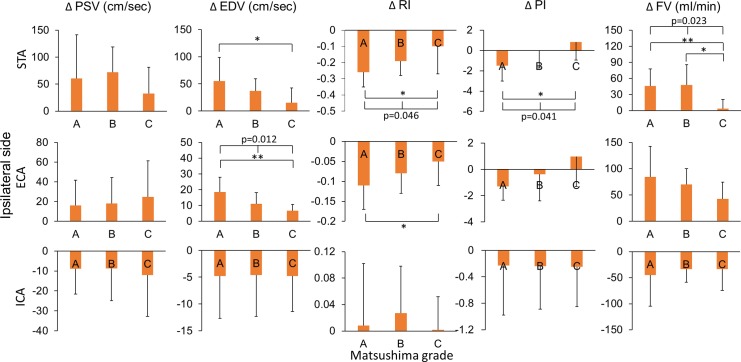
Relationship between Matsushima grades and postoperative ultrasonographic changes of the ipsilateral extracranial arteries. Matsushima grades were correlated with the post-operative changes in EDV, RI, PI, and FV of ipsilateral STA and ECA. (STA, superficial temporal artery; ECA, external carotid artery; ICA, internal carotid artery; ΔPSV, post-operative change of peak-systolic velocity; ΔEDV, post-operative change of end-diastolic velocity; ΔRI, post-operative change of resistance index; ΔPI, post-operative change of pulsatility index; ΔFV, post-operative change of flow volume; *, <0.05; **, <0.01).

**Fig 3:**
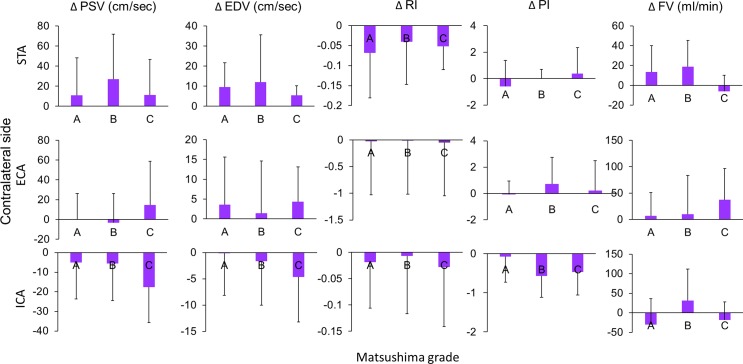
Relationship between Matsushima grades and postoperative ultrasonographic changes of the contralateral extracranial arteries. Matsushima grades were not correlated with the post-operative changes in all of the parameters of the contralateral STA, ECA, and ICA.

Considering the ultrasonographic changes between pre- and post-operative states may be a more important indicator for collateral establishment, we investigated the correlation between the postoperative changes (changes between post-operative and pre-operative values) of ultrasonographic parameters and the Matsushima grades. This analysis showed that postoperative changes of the ipsilateral STA and ECA were correlated with Matsushima grades (Fig 2). By comparison among the three Matsushima groups, Matsushima grades of better collateral establishment were correlated with a larger reduction in the RI and PI (p = 0.046 and 0.041, respectively) with a larger increase of flow volume (p = 0.023) of STA, and a larger increase in the EDV of ECA (p = 0.012) on ultrasonography. The postoperative hemodynamic changes in STA, ECA and ICA on the contralateral side had no correlation with Matsushima grades of the operative side (Fig 3).

**Fig 4:**
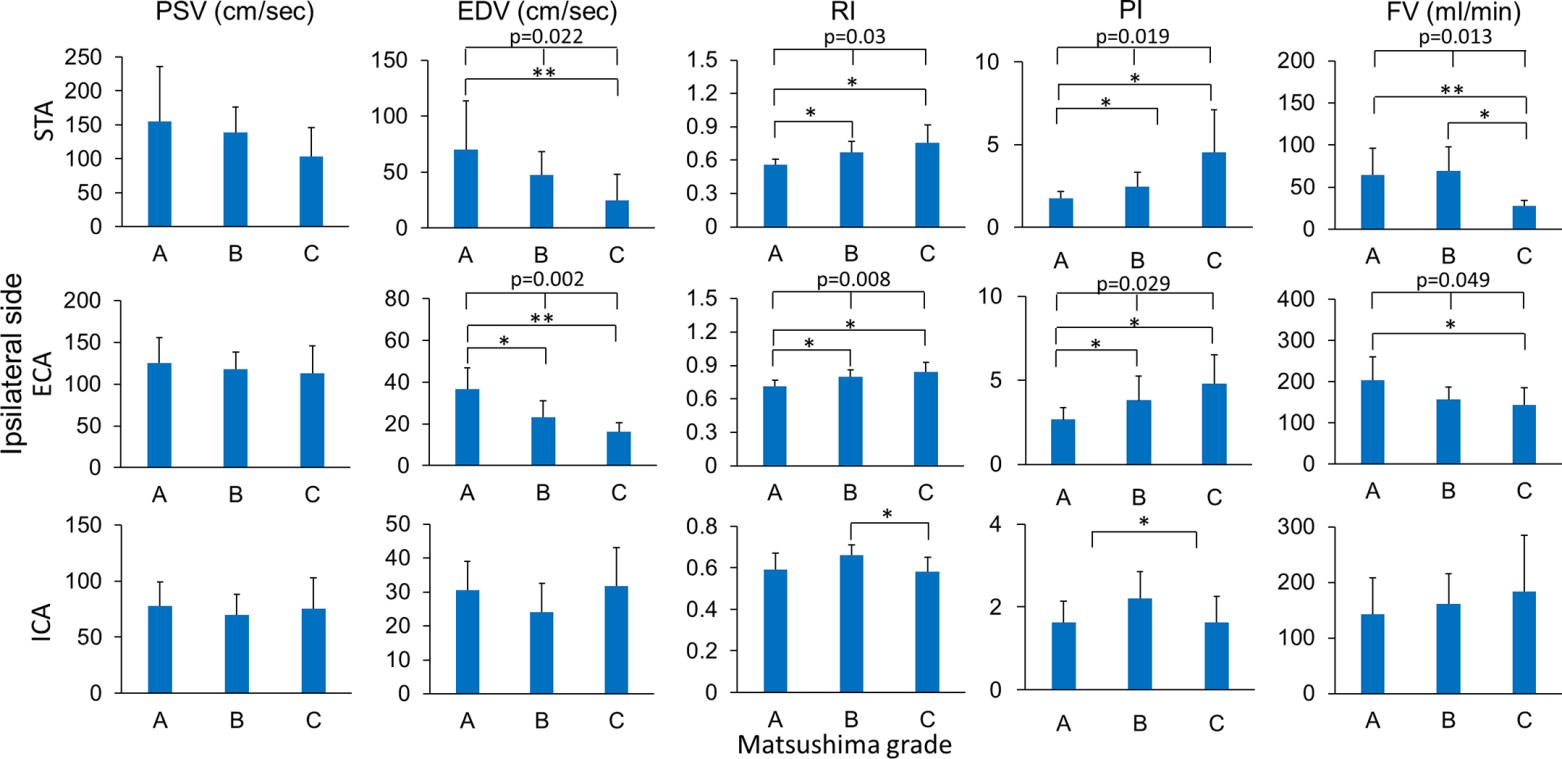
Relationship between Matsushima grades and postoperative ultrasonographic parameters of the ipsilateral extracranial arteries. Matsushima grades were correlated with the postoperative EDV, RI, PI, and FV of ipsilateral STA and ECA (*, <0.05; **, <0.01).

**Fig 5:**
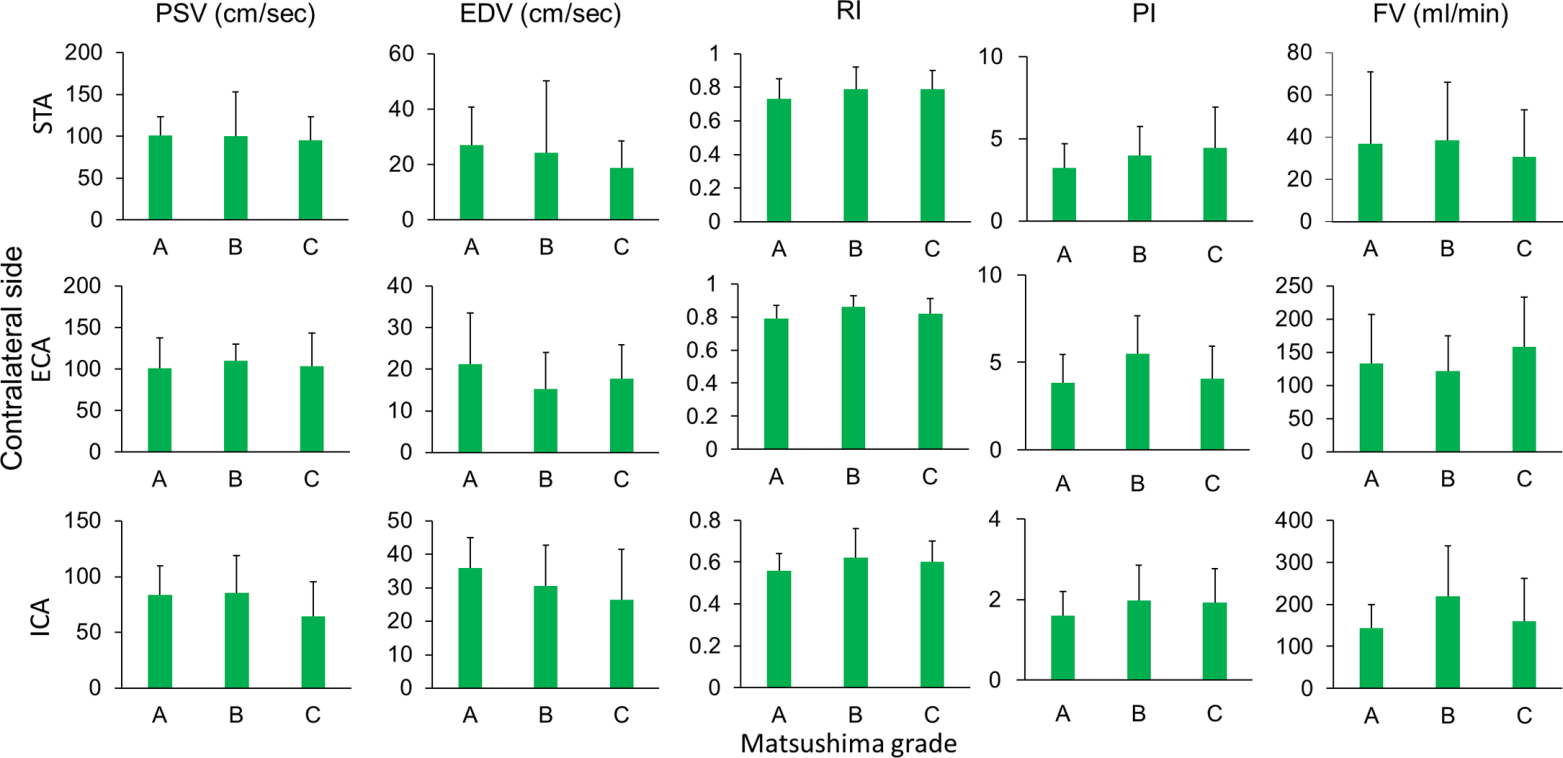
Relationship between Matsushima grades and postoperative ultrasonographic parameters of the contralateral extracranial arteries. Matsushima grades were not correlated with all of the post-operative parameters of the contralateral STA, ECA, and ICA.

The Matsushima grades also correlated with post-operative ultrasonographic parameters of STA and ECA, as shown in Fig 4. The comparison among the three Matsushima groups showed that better Matsushima grades also had a correspondingly higher EDV (p = 0.022 for STA; p = 0.002 for ECA), a lower RI (p = 0.03 for STA; p = 0.008 for ECA), a lower PI (p = 0.019 for STA; p = 0.029 for ECA), and a larger flow volume (p = 0.013 for STA; p = 0.049 for ECA) of the ipsilateral STA and ECA on the ultrasonographic study. The ICA on the ipsilateral side showed no significant intergroup difference among different Matsushima grades except for lower RI in the grade C group compared to the grade B group (0.58 ± 0.07 vs. 0.66 ± 0.05, p = 0.044). The ultrasonographic parameters of STA, ECA and ICA on the contralateral side had no correlation with Matsushima grades of the operative side (Fig 5). Furthermore, logistic regression analysis was performed to investigate the correlation of ultrasonographic parameters with Matsushima grades (Table 2). The EDV of ECA had a significant correlation with Matsushima grade A+B before adjustment (OR = 1.28, p = 0.024, 95% CI = 1.03–1.59), and after adjustment with age (OR = 1.47, p = 0.035, 95% CI = 1.03–2.11). The PI of STA and ECA also had significant correlation with Matsushima grade A+B before adjustment (STA: OR = 0.35, p = 0.035, 95% CI = 0.13–0.93; ECA: OR = 0.44, p = 0.034, 95% CI = 0.21–0.94) and after adjustment with age (STA: OR = 0.33, p = 0.026, 95% CI = 0.12–0.87; ECA: OR = 0.29, p = 0.024, 95% CI = 0.1–0.85). After adjustment with age, Suzuki’s stage, and operative procedures, the PI of STA still significantly correlated with Matsushima grade A+B (OR = 0.3, p = 0.046, 95% CI = 0.09–0.98).

**Table 2:** Odds ratios and 95% confidence intervals for association of ultrasonographic parameters with matsushima grade A+B on angiography.

	Unadjusted model			Adjusted model 1*			Adjusted model 2ǂ		
	OR	95% CI	p-value	OR*	95% CI*	p-value*	ORǂ	95% CIǂ	p-valueǂ
STA									
PSV	1.02	0.99–1.04	0.141	1.02	0.99–1.04	0.197	1.02	0.99–1.04	0.255
EDV	1.07	1–1.15	0.053	1.07	1–1.15	0.063	1.07	1–1.14	0.065
RI	–	–	–	–	–	–	–	–	–
PI	0.35	0.13–0.93	0.035	0.33	0.12–0.87	0.026	0.3	0.09–0.98	0.046
FV	1.05	0.99–1.33	0.064	1.17	0.98–1.4	0.081	1.2	0.95–1.52	0.117
ECA									
PSV	1.01	0.98–1.05	0.479	1	0.96–1.05	0.829	1.01	0.96–1.06	0.809
EDV	1.28	1.03–1.59	0.024	1.47	1.03–2.11	0.035	–	–	–
RI	–	–	–	–	–	–	–	–	–
PI	0.44	0.21–0.94	0.034	0.29	0.1–0.85	0.024	–	–	–
FV	1.02	1–1.05	0.102	1.03	1–1.06	0.077	1.03	0.99–1.06	0.119
ICA									
PSV	1	0.96–1.04	0.939	0.96	0.9–1.03	0.282	0.97	0.90–1.04	0.398
EDV	0.96	0.87–1.06	0.439	0.9	0.79–1.03	0.131	0.91	0.76–1.08	0.277
RI	–	–	–	–	–	–	–	–	–
PI	2.02	0.35–11.68	0.431	1.77	0.29–10.71	0.533	1.19	0.14–9.91	0.872
FV	0.99	0.98–1.01	0.321	0.99	0.97–1	0.168	0.99	0.97–1.01	0.273

*Adjusted model 1: Adjusted with age

ǂ Adjusted model 2: Adjusted with age, pre-operative Suzuki stage, and operation procedure

OR indicates odds ratio; CI, confidence interval

**Fig 6:**
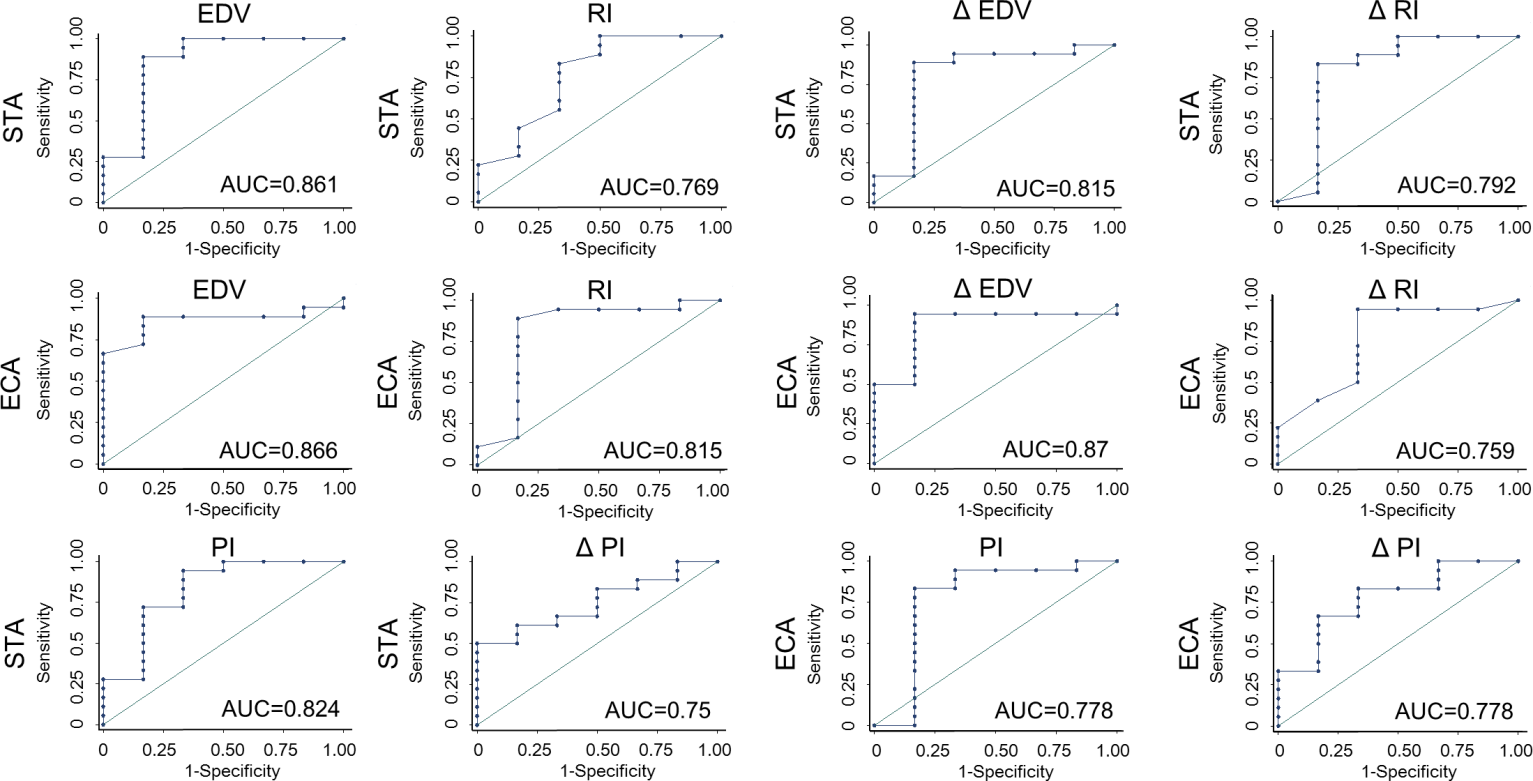
ROC analysis for prediction of Matsushima grade A+B by the ultrasonographic parameters of STA and ECA. The area under the curve of ROC (AUC) of each parameter was also shown, ranging from 0.75–0.87.

In addition, we sought to determine the cut-off points of the hemodynamic parameters on ultrasound corresponding to well-developed collateralization (Matsushima grade A+B) by Receiver Operating Characteristics (ROC) analysis. Fig 6 showed the results of ROC analysis for prediction of Matsushima grade A+B by the EDV, RI, PI, ΔEDV (changes between post- and pre-operative EDV), ΔRI (changes between post- and pre-operative RI) and ΔPI (changes between post- and pre-operative PI) in the ECA and STA, and the area under the curve of ROC (AUC) of each parameter was also shown (0.75–0.87). Table 3 showed the sensitivity, specificity, positive predictive value, and negative predictive values of ultrasonographic parameters in prediction of Matsushima grades A+B. The positive predictive values of post-operative EDV, RI, PI, ΔEDV, ΔRI and ΔPI in ECA and STA were between 0.86–1 in prediction of Matsushima grade A+B. Of note, post-operative increase of EDV greater than 13.5 cm/sec or reduction of RI greater than 0.19 in STA corresponded to 94% of Matsushima grade A+B. Moreover, post-operative EDV of ECA greater than 22 cm/sec or post-operative increase of EDV greater than 6.4 cm/sec in ECA also corresponded to 94% of Matsushima grade A+B.

**Table 3:** Prediction effect of the ultrasonic hemodynamic parameters of STA and ECA to Matsushima grades A+B.

		Matsushima A+B				
		Cut-off	SEN	SPE	PPV	NPV
STA	EDV	19 cm/sec	1.00	0.67	0.90	1.00
	RI	0.78	1.00	0.50	0.86	1.00
	PI	2.1	0.72	0.83	0.93	0.5
	ΔEDV	+13.5 cm/sec	0.89	0.83	0.94	0.71
	ΔRI	-0.19	0.83	0.83	0.94	0.63
	ΔPI	-1.3	0.5	1	1	0.4
ECA	EDV	22 cm/sec	0.89	0.83	0.94	0.71
	RI	0.82	0.89	0.67	0.89	0.80
	PI	3.7	0.83	0.83	0.94	0.63
	ΔEDV	+6.4 cm/sec	0.94	0.83	0.94	0.83
	ΔRI	-0.05	0.94	0.67	0.89	0.80
	ΔPI	-0.11	0.67	0.83	0.92	0.45

STA indicates superficial temporal artery; ECA, external carotid artery; EDV, end-diastolic velocity; RI, resistance index; PI, pulsatility index; ΔEDV, changes between post-operative and pre-operative EDV values; ΔRI, changes between post-operative and pre-operative RI values; ΔPI, changes between post-operative and pre-operative PI values; SEN, sensitivity; SPE, specificity; PPV, positive predictive value; NPV, negative predictive value.

**Fig 7:**
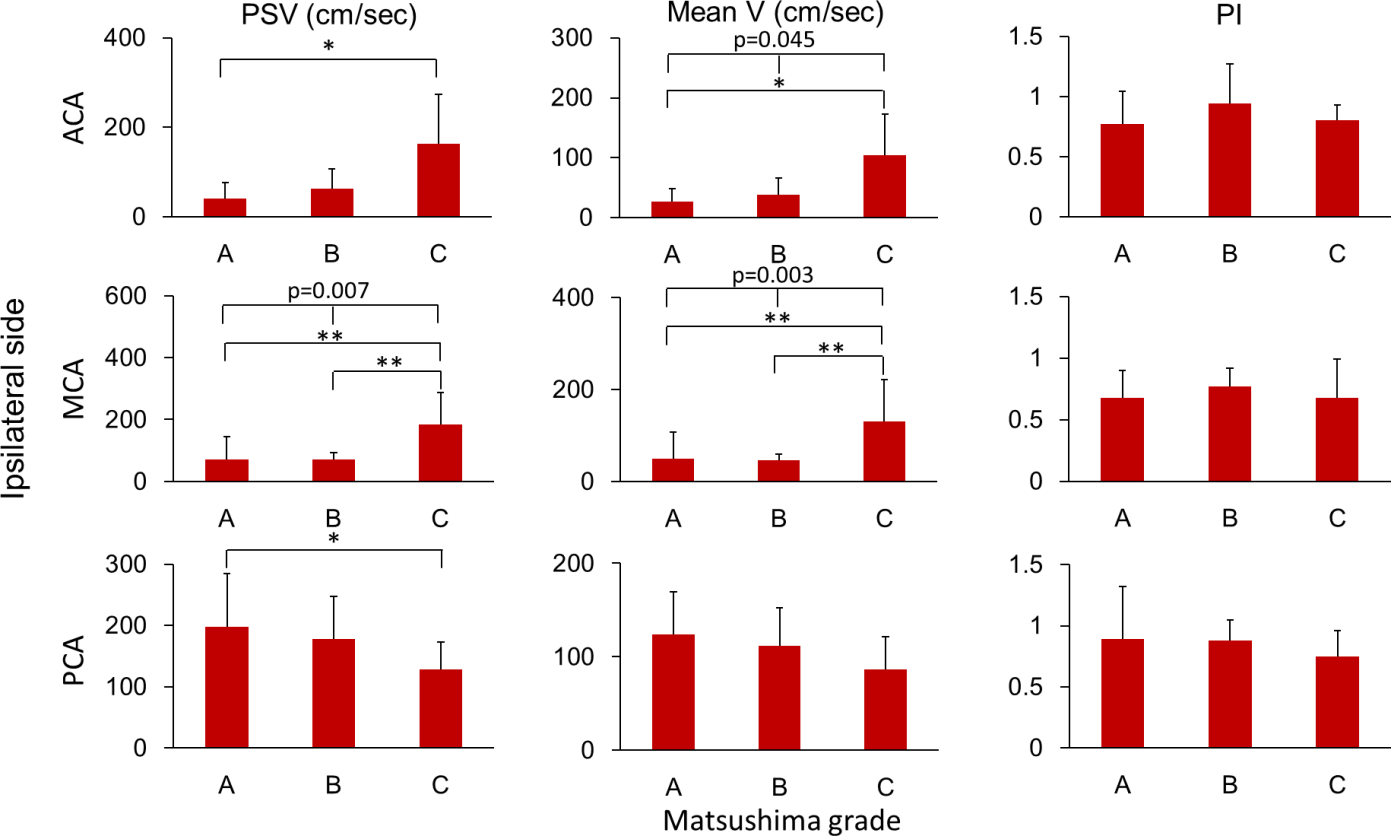
Relationship between Matsushima grades and postoperative ultrasonographic parameters of the ipsilateral intracranial arteries. Matsushima grades were correlated with the postoperative velocities of ipsilateral ACA, MCA and PCA. (ACA, anterior cerebral artery; MCA, middle cerebral artery; PCA, posterior cerebral artery; PSV, peak-systolic velocity; mean V, mean velocity; PI, pulsatility index; *, <0.05; **, <0.01.).

**Fig 8:**
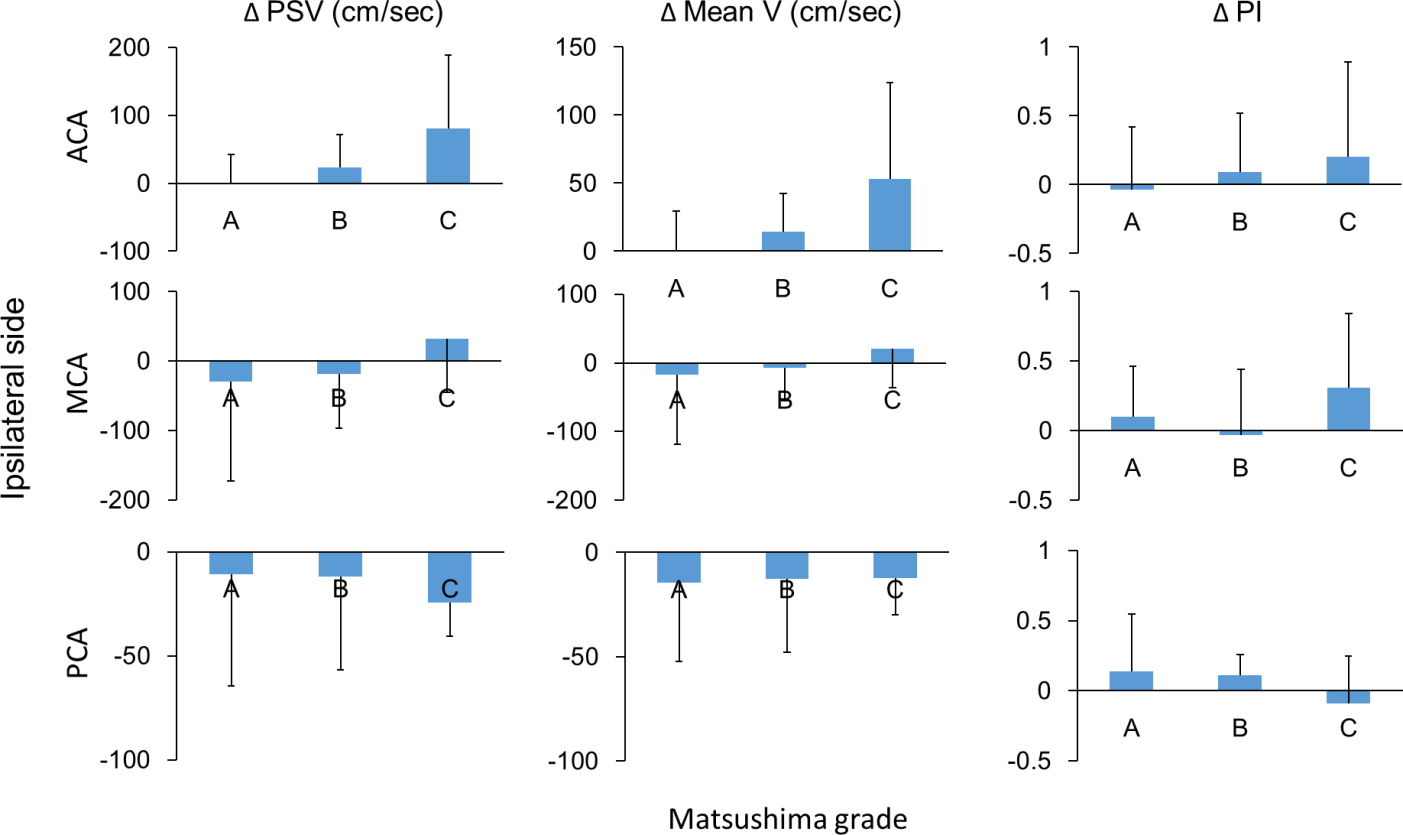
Relationship between Matsushima grades and postoperative changes of the ipsilateral intracranial arteries. Matsushima grades were not correlated with all the postoperative changes in the intracranial arteries.

Furthermore, the hemodynamic patterns of the intracranial arteries were shown in Fig 7, and the post-operative changes of these hemodynamic parameters were presented in Fig 8. The flow velocity of MCA in the groups of Matsushima A and B were at lower border of normal limits (mean velocities of group A and B were 50.4 cm/sec and 46.9 cm/sec, respectively), while it was much higher in the group of Matsushima C (mean velocity: 131.3 cm/sec; p between group A and C: 0.005; p between group B and C: 0.005; p among three groups = 0.003). The flow velocity of MCA was decreased compared to baseline in the groups of Matsushima A and B (Δmean velocities of group A and B were -17.6 cm/sec and -7.6 cm/sec, respectively). The flow velocity of ACA was highest in Matsushima C group and lowest in Matsushima A group (mean velocities of C and A groups were 104.3 cm/sec and 26.6 cm/sec, respectively; p between group A and C: 0.045; p among three groups = 0.045), and the flow velocity of ACA was increased compared to baseline in the groups of Matsushima B and C especially in group C (Δmean velocities of group B and C were +14.1 cm/sec and +53.2 cm/sec, respectively). The PI of the ACA was increased in Matsushima C groups (ΔPI of group C: +0.2) but without significant intergroup difference. In PCA, even though there was mildly drop of flow velocity compared to baseline (Δmean velocities were -14.5cm/sec, -12.9 cm/sec, and -12.3 cm/sec in groups A, B and C, respectively), the flow velocities of PCA remained higher than normal in the three groups (highest in group A, 197.4 cm/sec; lowest in group C, 127.7 cm/sec; p between group A and C: 0.044).

## Discussion

This study showed that moyamoya patients who had better neovascularization on postoperative angiography were found to have higher flow velocity, lower flow resistance, and larger flow volume in STA and ECA on postoperative color Doppler ultrasound. The postoperative changes in ultrasonographic parameters of STA and ECA compared to pre-operative state were also correlated with Matsushima grades. These findings demonstrated that these parameters of STA and ECA on color Doppler ultrasound have parallel changes with angiography in terms of collaterals.

Transdural and transpial collateral establishment is a key marker that ought to be evaluated for moyamoya patients after indirect revascularization operation. Indirect revascularization procedures can induce neoangiogenesis into the leptomeningeal vascular system by relocating autologous tissues such as vessels, dura, temporalis muscles, periosteum, or galea on the brain surface [6]. After indirect revascularization surgery, increased regional CBF has been demonstrated on Xenon-enhanced computed tomography (CT) scan, perfusion CT, perfusion magnetic resonance image, and single-photon emission CT studies [4, 15, 16]. Furthermore, the extent of neovascularization from the graft tissue was parallel to the changes in regional CBF [17]. Moreover, Cho et al. [18] showed that the degree of neovascularization on cerebral angiographic images after indirect bypass surgeries correlated with clinical outcome. In cases of inadequate revascularization with symptoms of cerebral hypoperfusion, second salvage surgery should be considered. Therefore, an easy and noninvasive tool for evaluation of neovascularization after indirect revascularization surgery will be useful for follow-up of moyamoya patients.

Few published reports compared ultrasonographic and angiographic findings with respect to neovascularization by color Doppler and power Doppler imaging of ultrasound in moyamoya patients. A case report revealed the intracranial hemodynamic behaviour of MMD after non-aneurysmal SAH by using serial transcranial color Doppler [12]. It showed progressively lowering of resistance in the right MCA, as well as consistently high velocity with low resistance in the remaining intracranial arteries. The cerebral angiography revealed abundant moyamoya vessels branching from the right MCA and rich intracranial collaterals from the left STA and ECA, which suggested that the hemodynamic behaviour of the intracranial arteries on ultrasound gradually changed from the influence of SAH to the features influenced by the collateral network of MMD [12, 19]. In other case series studies, Perren et al. [10, 13] showed that the grading of neovascularization after EMS had significant agreement between power Doppler imaging and angiography. The present study used color Doppler imaging to show a significant correlation between ultrasonic hemodynamic parameters and angiographic Matsushima grades in moyamoya patients after indirect revascularization surgery, both reflecting the degree of transdural and transpial collaterals from STA and ECA. In the velocity parameters, the differences in EDV among different Matsushima grades were statistically significant, while those in PSV were not. In addition, the RI and PI also showed significant inter-group differences. Even after adjustment with age in the logistic regression analysis, EDV of ECA as well as PI of STA and ECA correlated significantly with Matsushima grades A+B. Therefore, color Doppler imaging of ultrasound may be a useful tool for follow-up of moyamoya patients after indirect revascularization surgery. We also proposed cut-off points of post-operative values and changes of EDV and RI in STA and ECA which may be helpful for clinical interpretation, but further validation with more cases is needed.

There were several interesting findings in the intracranial hemodynamic patterns according to different Matsushima grades. The groups with good post-operative neovascularization (Matsushima A and B) had concomitantly slower velocity in the MCA compared to baseline especially in the group A, while those with poor neovascularization (Matsushima C) had much higher flow velocity in the MCA. In addition, the pre-operative Suzuki stage was 2 (initiation of moyamoya) in Matsushima C group while 3 (intensification of moyamoya) in Matsushima A and B groups. These two findings suggested that the stenosis of the MCA in the group of Matsushima C was less severe than that in Matsushima A and B; therefore, the induction of neovascularization was weaker in the Matsushima C group. Furthermore, the flow velocities of PCA were all higher than normal but mildly dropped compared to baseline in the three Matsushima groups, with increased PI in Matsushima A and B groups while decreased PI in the C group compared to baseline, meaning the compensatory flow from PCA to anterior circulation was decreased after operation in Matsushima A and B groups while this compensatory flow remained important in Matsushima C group.

This study enrolled adult and pediatric moyamoya patients with variation in the follow-up duration. Pediatric patients have been proved to have better collateral establishment after indirect revascularization surgery than adult patients [20]; therefore, we performed the adjustment of age in the logistic regression model. Besides, the hemodynamic changes after indirect revascularization surgery gradually developed in several months [21], so patients at different post-operative period may have different amount of neovascularization. However, this present study focused on the comparison between angiographic and ultrasonographic patterns; therefore, we limited the time interval between the two examinations to ensure that the two examinations were done within the same state of neovascularization in each patient.

There are several limitations in this study. First, color Doppler imaging cannot directly quantify the amount of neovascularization. Although it provided indirect information about the amount of revascularization, its hemodynamic parameters were proved to have significant correlation with angiographic Matsushima grades in this study. Second, we enrolled patients who received more than one kind of indirect revascularization surgery; however, all of these surgeries developed neovascularization through the same mechanism of pial synangiosis, and used STA as a material for neovascularization. Third, intra-observer variation was a potential problem for color-Doppler ultrasound examination. We measured each parameter once if the waveform was stable because many patients were children who could not tolerate prolonged examinations, and the diameter was measured at end-diastole to minimize intra-observer variation [21]. Fourth, the case number was small because MMD was a rare disease. Further validation for our findings in a larger case number is mandatory in the future. The strength of this study is that it provided evidence for the first time that the angiographic collateral grades correlated well with the hemodynamic parameters on color-coded ultrasound after indirect revascularization surgery.

## Conclusions

After indirect revascularization surgery in moyamoya patients, color Doppler ultrasonographic findings in the ipsilateral STA and ECA correlated well with the extent of neovascularization from the ECA as shown on angiography. This correlation with angiographic findings shows color Doppler ultrasonography to be a potential, less invasive postoperative evaluation tool which can reduce discomfort in patients during follow-up. For the patients with ultrasonographic findings indicating poor collateral establishment, angiography should be considered for determination of secondary salvage surgery.

## Supporting information

S1 Table Original ultrasonographic data of the cases in this study. (XLSX)
