# Correction: Color Doppler ultrasonography as an alternative tool for postoperative evaluation of collaterals after indirect revascularization surgery in Moyamoya disease

**DOI:** 10.1371/journal.pone.0191381

**Published:** 2018-01-11

**Authors:** The PLOS ONE Staff

The following information is missing from the Funding section: This study was supported by a grant from the National Science Council of the Republic of China (MOST 105-2314-B-002–004 -MY2) for Dr. Meng-Fai Kuo. The publisher apologizes for the error.
